# Shutdown of interferon signaling by a viral-hijacked E3 ubiquitin ligase

**DOI:** 10.15698/mic2017.11.600

**Published:** 2017-11-03

**Authors:** Kaitlin A. Davis, John T. Patton

**Affiliations:** 1 Department of Biology, Johns Hopkins University, Baltimore, MD 21218.; 2 Department of Biology, Indiana University, Bloomington, IN 47405.

**Keywords:** rotavirus, interferon, ubiquitin, E3 ligase, β-TrCP

## Abstract

Viruses manipulate cellular processes to create an environment favorable to replication. For most viruses, this includes subverting the expression of interferon (IFN), a signaling molecule that can stimulate production of a vast array of antiviral gene products. Rotavirus, a segmented double-stranded RNA virus that causes acute gastroenteritis in infants and young children, inhibits IFN expression through its nonstructural protein NSP1. This viral protein stifles IFN expression by inducing the degradation of host factors that are necessary for upregulating the activity of IFN genes. In the case of nearly all human and porcine rotavirus strains, NSP1 induces the ubiquitination-dependent proteasomal degradation of β-transducin repeat containing protein (β-TrCP), a host factor that plays an essential role in activating the IFN-transcription factor, NF-κB. Key to the process is the presence of a decoy sequence (degron) at the C-terminus of NSP1 that causes β-TrCP to mistakenly bind NSP1 instead of its natural target, inhibitor-of-κB (IκB). In a recent report published by Davis *et al* [2017; mBio 8(4): e01213-17], we describe molecular requirements that govern NSP1 recognition of β-TrCP, including an essential degron phosphorylation event, and the step-wise incorporation of NSP1 into hijacked cullin-RING E3 ligases (CRLs) that ubiquitinate and tag β-TrCP for degradation. Notably, although β-TrCP is chiefly recognized for its role as a master regulator of NF-κB signaling and IFN expression, β-TrCP also controls the stability of checkpoint proteins implicated in numerous other cellular pathways with antiviral activities, including autophagy and apoptosis. Thus, the impact of NSP1 on creating an intracellular environment favorable to virus replication may extend well beyond the IFN signaling pathway.

Viruses have evolved a number of mechanisms to combat host antiviral responses in order to establish a pro-viral cellular environment. Many host antiviral responses rely on signaling cascades initiated by the production of IFN. Rotavirus, a pathogen known to infect nearly all known mammalian and avian animal species, employs NSP1 to counter IFN production. NSP1 proteins encoded by various rotavirus strains share little sequence conservation except for the presence of a putative N-terminal RING domain and a C-terminal substrate-targeting domain. While the targeting domain of most human and porcine rotavirus NSP1 proteins mediates the recruitment of β-TrCP, the targeting domain of many animal strains (simian, murine, equine, etc.) mediates the recruitment of IFN-regulatory factors (e.g., IRF-3/-7). NSP1 binding to β-TrCP or IRF proteins is correlated with proteasomal degradation of these targets in the infected cell.

There is a growing body of data to suggest that NSP1 triggers the degradation of targets by hijacking a subset of E3 ubiquitin ligases: the cullin-RING ligases (CRLs). Hijacked CRLs are presumed to direct the ubiquitination and proteasomal degradation of NSP1-bound targets. CRLs are large modular complexes that are minimally comprised of a cullin scaffold protein (Cul1, 2, 3, 4a, 4b, 5, 7), a RING-domain containing protein (Rbx1, 2), and a substrate adaptor that directs the CRL to the target protein. Through multiple experimental approaches, results have been obtained indicating that NSP1 associates with components of CRLs, including Cul3, which has led to the hypothesis that NSP1 functions a substrate adaptor protein of a Cul3-CRL. A recent publication by Davis *et al* (2017) reveals the highly coordinated sequence of events necessary for NSP1 recruitment of β-TrCP and the integration of the NSP1-β-TrCP complex into hijacked CRL complexes.

**Figure 1:**
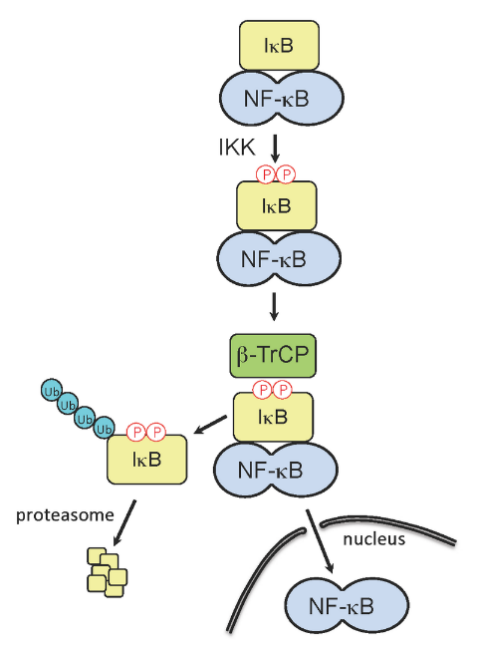
FIGURE 1: Activation of NF-κB. Upon detection of RNA-virus infection by the host cell, signaling cascades are induced which can trigger IFN expression and the establishment of an antiviral state. The inducible IκB kinase (IKK) plays critical role in IFN signaling cascades, as its activity is responsible for phosphorylation of the IκB degron. The phosphorylated IκB degron is recognized by β-TrCP, a substrate-specific adaptor of an E3 ubiquitin ligase complex, SCF^βTrCP^, leading to IκB ubiquitination and degradation. IκB degradation releases NF-κB, allowing the transcription factor to translocate to the nucleus and upregulate IFN genes.

NSP1 proteins that target β-TrCP have within their C-termini the sequence DSGXS. This sequence is a molecular decoy of the degron sequence, DSGφXS, contained in IκB. During activation of IFN signaling pathways, the IκB degron undergoes phosphorylation by the serine-threonine kinase activity of IκB kinase (IKK), creating a phosphodegron that is recognized by β-TrCP (Fig. 1). Interaction of β-TrCP with the IκB phosphodegron leads to IκB degradation, which in turn frees NF-κB to translocate to the nucleus and upregulate IFN promoter activity. In a parallel manner, binding of β-TrCP to the C-terminal IκB-like degron of NSP1 is dependent on phosphorylation. As shown by Davis *et al* (2017), this phosphorylation event is not only required for binding of β-TrCP to NSP1 but is also essential for the subsequent incorporation of the NSP1-β-TrCP complex into a CRL (Fig. 2). Unlike the degron of IκB, which is dependent on the inducible IKK for phosphorylation, the degron of NSP1 co-opts the use of the ubiquitously active serine-threonine kinase CKII for phosphorylation. Use of this kinase potentially offers several benefits to the virus. Most notably, the NSP1 degron is activated without the need for a kinase, like IKK, that is regulated through an immune signaling pathway. Moreover, CKII is associated with CRL accessory components (e.g. the COP9 signalosome), possibly placing NSP1 in the constant presence of an activating kinase. Through the use of NSP1 mutants and CRL inhibitors, Davis *et al* (2017) obtained data showing that CKII remains associated with the NSP1-β-TrCP complex even after degron phosphorylation, creating the metastable NSP1-CKII-β-TrCP complex. Thus, CKII may have a role in the subsequent assembly of NSP1-CRLs that extends beyond NSP1 phosphorylation and activation.

**Figure 2:**
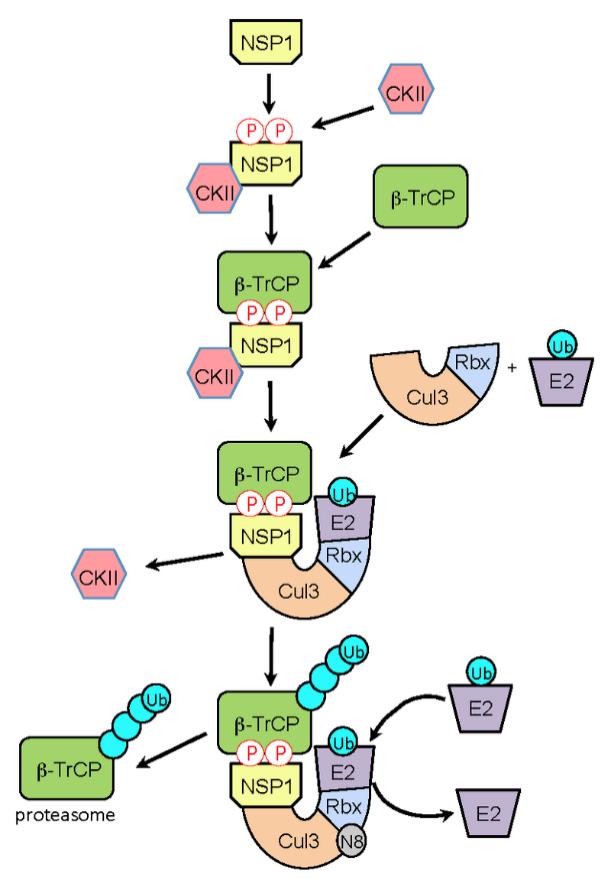
FIGURE 2: Model for NSP1-CRL3 assembly. Following expression in the infected cell, rotavirus NSP1 interacts with the constitutively-active kinase CKII, resulting in phosphorylation of NSP1 C-terminal IκB-like degron. The affinity of β-TrCP for the phosphorylated degron, leads to the formation of an NSP1-β-TrCP-CKII complex. CKII is released as the NSP1-β-TrCP complex interacts with Cul3-Rbx1 to form an NSP1-CRL3. Recruitment of a ubiquitin-charged E2 enzyme to the CRL, followed by neddylation (N8) of the cullin component, facilitates ubiquitination of the β-TrCP target. Successive rounds of ubiquitination generate a polyubiquitin chain that signals proteasomal degradation of β-TrCP.

In our Davis *et al* (2017) publication, we also present data indicating that the NSP1-CKII-β-TrCP complex interacts with Cul3 subunits, forming a Cul3-NSP1-β-TrCP complex. CKII is released at this step of the assembly process. The CRL3-NSP1-β-TrCP complex presumably binds a ubiquitin-charged E2 enzyme and its cullin component undergoes neddylation, initiating structural changes that facilitate transfer of ubiquitin to β-TrCP. Repeated cycles of binding and release of charged and uncharged E2s create a poly-ubiquitin chain on β-TrCP that is recognized by the proteasome. Interestingly, we found that mutation of the NSP1 RING domain (C42A) created a form of NSP1 that, although capable of binding β-TrCP and CKII, could not bind Cul3. This finding raises the possibility that phosphorylation of the NSP1 IκB-like degron triggers a structural change that allows interaction of the N-terminal RING domain with Cul3. Considered together, our analyses indicate that formation of NSP1-CRLs is a highly ordered process, initiated by CKII-phosphorylation, followed by binding of β-TrCP, and ending with the incorporation of NSP1-β-TrCP complexes into a CRL through an interaction requiring participation of the NSP1 RING domain.

β-TrCP has many cellular targets besides IκB. These targets similarly contain phosphodegron motifs, and many serve as checkpoint proteins for cellular pathways that mediate antiviral activities beyond IFN induction. As one example, β-TrCP is responsible for degrading DEPTOR, the protein that binds to and regulates the activity of mTOR, a protein kinase that plays a major role in autophagocytic and apoptotic responses. In addition, β-TrCP has roles in regulating levels of IFN receptor (IFNAR1) in IFN-treated cells, promoting maturation of the p105 precursor protein to the NF-κB p50 subunit, and processing procasepase-3 into the proapoptotic caspase 3. The diverse influence of β-TrCP on cellular pathways and antiviral responses suggests that viral proteins - like NSP1 - that prevent β-TrCP activity affect the entire intracellular milieu and may shift the cellular atmosphere at multiple levels in favor of virus replication. Indeed, the positive implications of attacking β-TrCP on virus replication may explain why other viruses, such as HIV, vaccinia virus, and Epstein Barr virus also target this protein. Continued efforts to establish how NSP1 antagonizes β-TrCP activity will not only lead to a better understanding of the basis of rotavirus pathogenesis, but also provide insight into the structure and function of CRLs and the role that β-TrCP plays in promoting an antiviral intracellular environment.

